# Is Heightened-Shoaling a Good Candidate for Positive Emotional Behavior in Zebrafish?

**DOI:** 10.3390/ani8090152

**Published:** 2018-08-24

**Authors:** Becca Franks, Courtney Graham, Marina A. G. von Keyserlingk

**Affiliations:** 1 Animal Welfare Program, Faculty of Land and Food Systems, University of British Columbia, Vancouver, BC V6T 1Z4, Canada; courtney3graham@gmail.com (C.G.); nina@mail.ubc.ca (M.A.G.v.K.); 2 Animal Studies, Department of Environmental Studies, New York University, New York, NY 10003, USA

**Keywords:** fish welfare, positive emotions, shoaling, affiliative behavior, zebrafish, positive welfare, group cohesion, behavioral synchrony, naturalistic housing, environmental enrichment

## Abstract

**Simple Summary:**

Despite increasing interest in fish welfare, we still know very little about positive emotions in fish. This study had two aims: (1) to characterize a previously undescribed social behavior in zebrafish, “heightened-shoaling”, and (2) to evaluate whether heightened-shoaling may be a good candidate for future research into positive emotional behavior in zebrafish. Observing six groups of zebrafish housed in 110 L tanks furnished with a sloping gravel substrate, rocks, and artificial plants (10 fish/tank), we found that heightened-shoaling is marked by tight group cohesion, high behavioral synchrony (all fish engaging in the same behaviors at the same time), and low agonism. Episodes of heightened-shoaling occurred in all six groups, but rarely at the same time (only once out of 31 episodes), suggesting that the onset of heightened-shoaling is driven by internal group dynamics rather than external influences. Heightened-shoaling also appeared to be self-reinforcing as it had sustained durations—typically lasting for over 7 min and sometimes lasting for nearly half-an-hour. Collectively, these results are similar to the patterns that typify positive emotional behavior in other animals, for example, social grooming and social play. As the first description of heightened-shoaling, this research extends our knowledge of the range of zebrafish social dynamics and suggests a promising area for future research on positive emotions in fish.

**Abstract:**

Zebrafish, a highly-social species of freshwater fish, are widely studied across many fields of laboratory science including developmental biology, neuroscience, and genomics. Nevertheless, as standard housing for zebrafish typically consists of small and simplistic environments, less is known about their social behavioral repertoire in more naturalistic settings. In particular, spontaneously occurring, socio-positive affiliative behaviors (e.g., social coordination and cohesion) that may be indicative of positive emotional experiences have rarely been reported or studied deliberately in zebrafish. Housing adult zebrafish (10 fish/tank) in large semi-natural tanks (110 L; *n* = 6) with sloping gravel substrate, rocks, and artificial plants, we observed a previously undescribed behavior: Distinct periods of spontaneous, synchronized, compact aggregations, what we call “heightened-shoaling”. This project aimed to quantify the characteristics of this distinctive behavior and compare parameters of heightened-shoaling to baseline periods (normal behavior) and pre-feed periods (feed-anticipatory behavior). First, across 4 days, we selected video-clips (100 s each) from within (i) instances of heightened-shoaling (*n* = 9), (ii) baseline periods (*n* = 18), and (iii) pre-feed periods (*n* = 18). For each of these video clips, we scan sampled every 10 s to determine fish orientations and location within the tank and agonistic behavior. Next, we used an all-occurrence sampling method to record the timing and duration of all episodes of heightened-shoaling behavior when tank-lights were on (8:00 h to 18:00 h) across 10 days. From the scan-sampling data, we found that compared to baseline periods, heightened-shoaling was characterized by increased shoal cohesion (*p* < 0.0001), increased adherence to the horizontal plane (*p* < 0.0001), decreased agonism (*p* < 0.0001), and no diving behavior (lower positions within the water column signal negative effect in zebrafish, *p* > 0.1). From the all-occurrence sampling data, we found 31 episodes of heightened-shoaling with instances observed in all six tanks and only a single case in which heightened-shoaling occurred in two tanks at the same time. The median episode duration was 7.6 min (Range 1.3–28.6). As the first systematic description of heightened-shoaling behavior, this research contributes to our knowledge of the range of zebrafish social dynamics living in naturalistic environments. Moreover, as a spontaneously occurring, protracted, affiliative behavior, heightened-shoaling appears to be a good candidate for future research into positive emotional behavior in zebrafish.

## 1. Introduction

In the past few decades, zebrafish (*Danio rerio*) have become increasingly used in many areas of laboratory science including developmental biology, neuroscience, and genomics [1]. Typical housing for zebrafish in laboratories consists of 3 L, barren and transparent recirculating tanks suspended on racks. Research on zebrafish welfare, however, has shown that zebrafish have preferences for more complex environments that contain, for example, gravel substrate and plants [2,3]. Housing animals in environments that they find to be aversive can lead to poor welfare [4], raising the possibility that zebrafish in standard laboratories may have poor welfare including altered behavioral repertoires and suboptimal affective states, such as increased negative emotions and reduced positive emotions. Housing zebrafish in relatively spacious (110 L), naturalistic tanks (i.e., with sloping gravel substrate, plants, and rocks), we observed a behavior that has not be described elsewhere in the scientific literature: Discrete periods of markedly tight group cohesion and increased behavioral synchrony; we termed this behavior “heightened-shoaling”. Given that heightened-shoaling was observed under these semi-natural conditions but appears to be absent from the extensive literature on zebrafish housed in barren tanks, one intriguing possibility is that it may be a candidate for future research into positive emotional experience in zebrafish. 

While negative emotions have attracted a substantial amount of zebrafish research—for example, studies on pain [5], fear [6], anxiety [7], stress [8], and depression [9,10]—positive emotions have rarely been the object of deliberate scientific inquiry in fish. Across several disciplines—e.g., psychology [11] and animal behavior [12,13]—positive emotions are increasingly recognized as distinctive contributors to emotional experience and as such, fundamental to the study of any particular organism’s behavioral biology and welfare. To make headway in understanding positive emotions in fish, feed anticipation and propensity to engage in the exploration of novel areas have been identified as promising areas of inquiry [14]. Under free-choice conditions, exploration is typically rewarding [15], and recent work has shown that zebrafish show heightened group cohesion and coordination in response to free-choice exploration opportunities [16]. Thus, there are theoretical and empirical reasons to associate increased group cohesion with positive emotional experience in zebrafish. However, zebrafish are also known to increase group cohesion and polarity in response to threats [17], making the valence of increased cohesion ambiguous on its own. 

Across much of the animal kingdom, threats are known to increase cohesion and affiliative behavior more generally (reviewed in References [18,19]). Nevertheless, social affiliation is thought to be one of the functional aspects of positive emotion insofar as social bonds are central to survival in many species (reviewed in Reference [14]). Moreover, the link between prosocial affiliative behavior and positive emotions has been demonstrated empirically. In humans, behavioral synchrony increases positive emotions, and mimicking or matching behavior is more likely to occur when people are in positive moods (reviewed in Reference [19]). Similar patterns linking affiliative behavior and positive emotions have also been found in nonhuman animals. For example, in fish, tactile stimulation can lower stress levels [20] and facilitate future prosocial interactions [21]. In nonhuman animals, social play is perhaps the most commonly studied affiliative behavior associated with positive emotions. Social play is characterized by being highly contagious, attracting non-participatory members into the activity, and tends to be self-sustaining, lasting for protracted periods of time without extraneous intervention [22]. Overall, therefore, these patterns suggest that in the absence of a threatening external stimulus or overt signs of distress, spontaneous, synchronized, protracted social affiliation is likely to involve positive emotion [23]. 

To improve our understanding of heightened-shoaling and to assess its ability to meet these criteria, we sought to quantify the distinctive behavioral features of heightened-shoaling and collect preliminary data on its frequency, timing, and duration. The goal of this research was, thus, twofold. First, to expand our knowledge of the range of zebrafish social dynamics, we aimed to systematically characterize the features of this previously undescribed behavior. Second, we aimed to determine whether the characteristics of heightened-shoaling were consistent with the hallmarks of positive emotional behavior found in other species, which, if they were, would indicate that heightened-shoaling would be a reasonable candidate for future research into positive emotional experience in zebrafish. We hypothesized that if heightened-shoaling appeared to be spontaneous (i.e., not caused by some room-level event), synchronized within tank, protracted, and without signs of negative affect (diving behavior in zebrafish [24]), this behavior would be a worthwhile measure to consider for future research on positive emotional behavior in zebrafish. 

## 2. Methods

### 2.1. Animals and Housing

**Figure 1:**
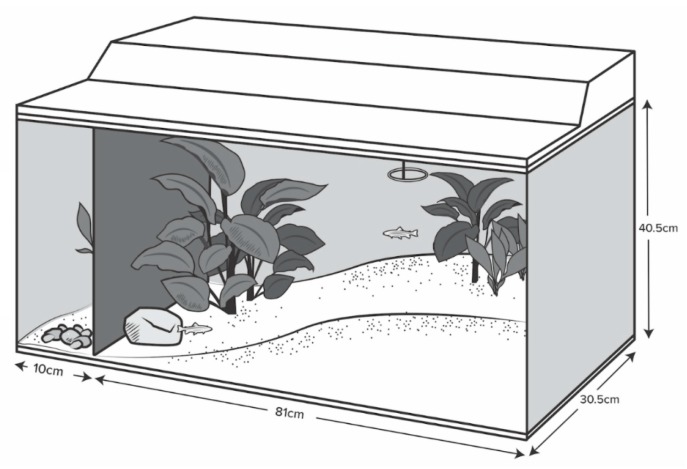
Diagram of 110 L semi-natural tanks. Circle at top right indicates location of the feeding ring (adapted from Reference [16]). Note inaccessible area at left (10 cm wide).

Wild-type adult zebrafish, all approximately 3 cm in length, were obtained from a local pet store (Aquariums West, Vancouver, BC, Canada) in August 2014 and estimated to be three to four months of age. From arrival until data collection began in February 2015, fish were housed in mixed-sex groups of 10 within glass tanks (110 L; 91 × 30.5 × 40.5 cm; *n* = 6) filled with conditioned water maintained at 26–28 °C. Tanks were furnished with a sloped gravel substrate, rocks, and five artificial plants placed to create a structurally complex environment (Figure 1). All tanks faced the same wall and opaque black plastic covered the back and side-walls of the tank to prevent visible interaction with other tanks and to minimize exposure to the room. A small area at the end of each tank was left inaccessible for future experimental needs (See Reference [16]; Figure 1). Video cameras (Swann NVR8-7200; resolution: 1000 TVL/1080p, Swann Communications, Melbourne, Australia), fastened to the front of each tank located approximately 1 m from the glass, continuously recorded fish behavior.

Room lights turned on at 7:00 h and turned off at 21:00 h, with tank lights turning on at 8:00 h and off at 18:00 h to create a semi-graduated light intensity change. Fish were fed a diet of flake food (Nutrafin Max Tropical Fish Flakes, Hagen, Baie D’Urfeé, QC, Canada) in the mornings at 10:00 h and thawed frozen blood worms (Hikari Bio-Pure, Hayward, CA, USA) in the afternoons at 16:00 h; fish were fed to satiation (food delivered in small pinches until food remained on the surface for more than 3 s) and both meals were delivered through a standard feeding ring in the shallow end of the tanks (Figure 1). Every week, 20% of the tank water was changed and water condition—pH, ammonia, nitrate, nitrite, carbonate hardness, general hardness, and dissolved oxygen—was tested two days after water changes to assure adequate water quality. All experimental and husbandry procedures were approved by the University of British Columbia’s Animal Care Committee (protocol A14-0119). 

### 2.2. Scan Sampling

Scan samples of fish behavior were collected to determine the behavioral features that distinguished heightened-shoaling. Accordingly, we examined behavior from three different period types: baseline, pre-feeding, and heightened-shoaling. The pre-feeding period started when the door to the room was opened and ending when the fish were fed. The baseline period was any time when the tank lights were on (8:00 h to 18:00 h) outside of feeding times (which we defined as beginning when the caregiver opened the door and ending five minutes after the last fish had finished feeding). The heightened-shoaling periods were clearly identifiable as protracted durations of tight group cohesion, with high participation rates (all fish in the tank) and increased behavioral synchrony (i.e., high group swimming coordination). While the fish were occasionally highly cohesive or synchronized for brief periods of time (e.g., 1–2 s) during baseline or ‘normal’ behavior, informal observation suggested the heightened-shoaling lasted for substantially longer durations and was categorically different. The goal of the present research was, therefore, to quantify the behavioral parameters of heightened-shoaling to characterize it systematically and empirically. See supplementary material for videos of behavior from each period type. 

Across 4 days between February through July 2015, we selected 18 baseline periods (3 per tank) and 18 pre-feed periods (3 per tank) and observed a total of 9 episodes of heightened-shoaling (during these 4 days, three tanks showed no heightened-shoaling, one tank had 2 episodes, one tank had 3, and one tank had 4). Within each period, we coded behavior during a 100 s video-clip (45 video clips in total). Every 10 s during the 100 s clip, observers trained to > 0.8 reliability noted: (i) The X-Y coordinates of all visible fish (X-Y coordinates recorded at the center of each fish), and (ii) the direction each fish was facing using clock coordinates (e.g., 9 o’clock is facing directly to the left, 1 o’clock is facing up and slightly to the right, etc.). Agonism was scored as either present or absent during the 10 s clips and was defined as a chase by an aggressor and a retreat by a recipient, a nip or bite at another fish, or a lateral display in which two fish swim parallel to each other in opposite directions and circle with their dorsal fins raised and caudal fins extended. All coders were blind to study predictions and hypotheses.

### 2.3. All-Occurrence Sampling

All-occurrence sampling was collected to determine the frequency, timing, and duration of heightened-shoaling. We selected 10 days between February and July 2015 for all-occurrence sampling to gather data on every instance of heightened-shoaling in all tanks during tank-light-on hours (8:00 h to 18:00 h), producing 600 hours of video data for analysis. Videos were viewed in VLC video media player (version 2.2.2 for Mac) using keyboard shortcuts that skipped video ahead in 10-s increments while maintaining visible fish outlines to allow for the identification of periods of heightened shoaling. The timing and duration of all such episodes were recorded for all 6 tanks. 

### 2.4. Statistical Analysis

To control for repeated observations within study days, tanks, periods, and scan-samples, we used multilevel modeling with random intercepts and Satterthwaite correction for degrees of freedom. Multilevel models of this kind are useful in that they can accommodate unequal sample sizes across conditions while also correcting for pseudoreplication [25,26]. For the scan sampling, we evaluated differences by period type (i.e., baseline, feeding, heightened-shoaling) for: (i) horizontal position, (ii) vertical position, with lower levels in the water column indicating distress in zebrafish [24], (iii) deviation from the horizontal plane, (iv) inter-fish distances (measured from the center of the fish), and (v) the presence/absence of agonism. More specifically, deviation from the horizontal plane was calculated by converting the clock-face orientation data into absolute degrees of deviation from the nearest horizontal plane. For example, if a fish were facing 1 o’clock, it would receive a deviation score of 60 (as would a fish facing 5, 7, or 11 o’clock). The presence/absence of agonism was modeled using a logit link and a Binomial error distribution (i.e., multilevel logistic regression). All other data were modeled using an identity link and a Gaussian error distribution (i.e., regular multilevel regression). For the all-occurrence sampling, we evaluated the typical duration of shoaling episodes, the start times, and co-occurrence of start times across tanks within the room. 

## 3. Results

### 3.1. Scan Sampling

**Figure 2:**
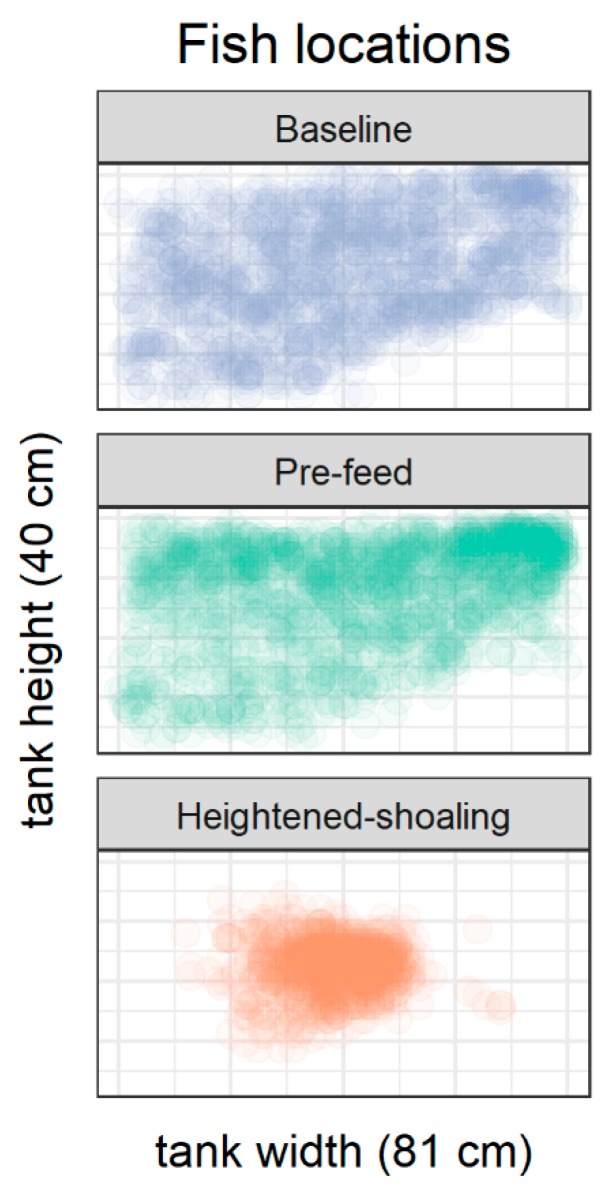
Location of each fish by period type. The X-Y coordinates as viewed from the side of a tank (to-scale, not including the inaccessible area, *n* = 6 tanks; 10 fish per tank). The location of each fish was collected from a snapshot taken every 10 s during the 100 s video clips (18 baseline clips, 18 pre-feed clips, 9 heightened-shoaling clips; 45 clips, 450 snapshots in all). Fish were higher in the water column pre-feed than the other two periods (baseline: *p* < 0.001; shoaling; *p* < 0.001), but otherwise their average location in the tank was indistinguishable across observation periods (*p* > 0.1). The absence of observed fish locations in the bottom right corner of each panel is explained by the presence of the gravel slope that angled up from the bottom middle of the tank to about halfway up the right side of the tank. Note also that the heightened shoaling episodes occurred at the center of the tank and in the middle of the slope.

For summary statistics of all behavioral parameters by observation period, please see Table 1. The *x*-axis location of the fish (left-right location when viewing the tank front-on) did not vary by observation period (all t’s < 1; all *p*’s > 0.64). The *y*-axis location—height in the water column—did vary, with the fish being at the same height during baseline and heightened-shoaling (t(36.50) = 1.52, *p* > 0.13) and higher in the water column during pre-feed compared to the other two periods (vs. baseline: t(23.50) = 3.83, *p* < 0.001; vs. heightened-shoaling: t(36.20) = 4.17, *p* < 0.001; Figure 2).

**Figure 3:**
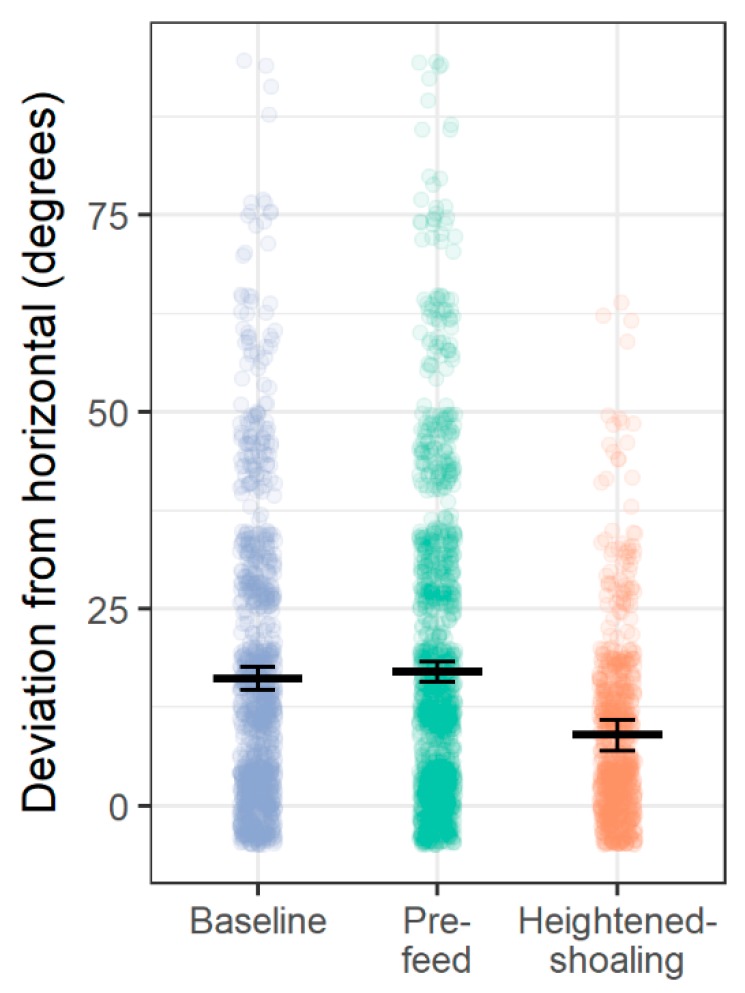
Deviation of each zebrafish from horizontal plane by period type. Small dots represent the orientation of a single zebrafish; dots are slightly jittered to avoid overlap (*n* = 6 tanks, 10 zebrafish per tank). Large horizontal lines and error bars correspond to multilevel model means and 95% confidence intervals.

**Figure 4:**
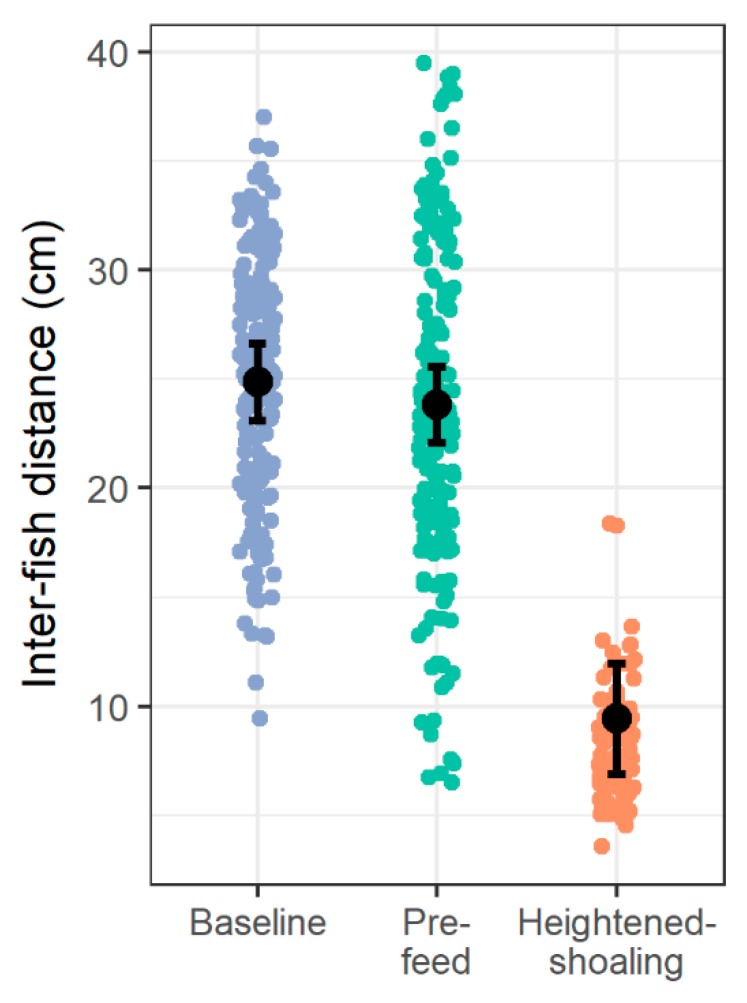
Inter-fish distances by period type. During heightened-shoaling, fish had significantly lower inter-fish distances compared to the other two observations periods (both *p*’s < 0.0001). Small dots represent average inter-fish distances during a snapshot of fish locations within a tank and are jittered slightly to avoid overlap (45 total clips, 450 snapshots; *n* = 6 tanks, 10 zebrafish per tank). Larger black dots and error bars correspond to multilevel model means and 95% confidence intervals. Note nearly non-overlapping distributions between heightened-shoaling and other periods.

**Figure 5:**
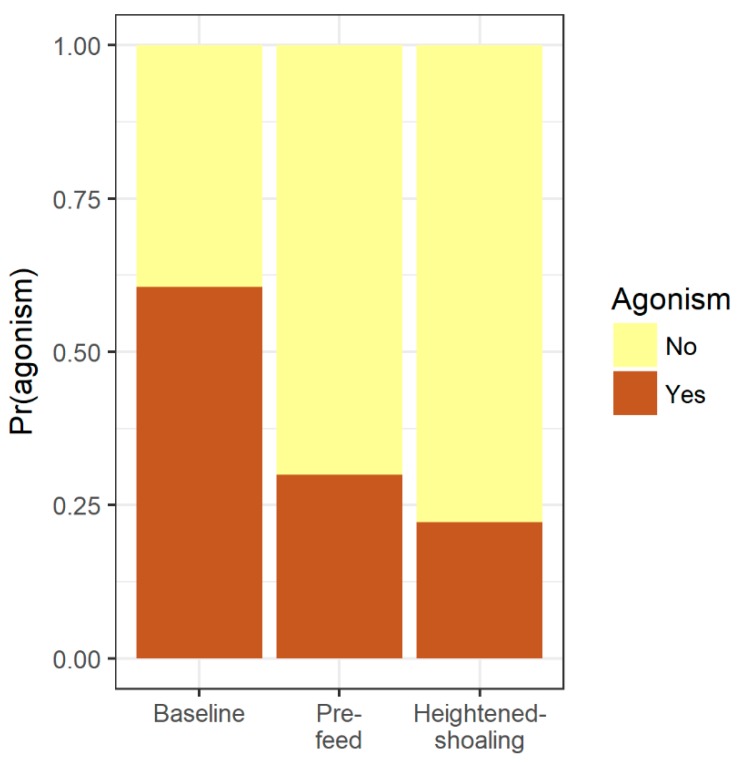
Probability of observing agonism during a ten-second window during each period type. Compared to baseline periods, zebrafish were significantly less agonistic during pre-feed and heightened-shoaling periods (both *p*’s < 0.0001; 45 video clips, 450 ten-second windows, *n* = 6 tanks, 10 zebrafish per tank).

Compared to baseline and pre-feed, during the heightened-shoaling periods, the fish adhered to the horizontal plane more consistently (vs. baseline: t(48.66) = 6.17, *p* < 0.0001; vs. pre-feed: t(38.90) = 7.36, *p* < 0.0001; Figure 3) and inter-fish distances were smaller (vs. baseline: t(43.60) = 10.73, *p* < 0.0001; vs. pre-feed: t(40.00) = 10.22, *p* < 0.0001; Figure 4). The highest levels of agonism were observed during baseline (61% of scan-samples), followed by pre-feeding (30% of scan-samples), and then heightened-shoaling (22% of scan-samples), with agonistic levels being significantly lower during pre-feed and heightened shoaling than during baseline (logit link model for heightened-shoaling: z = 3.90, *p* < 0.0001; pre-feed: z = 4.04, *p* < 0.0001; Figure 5).

### 3.2. All-Occurrence Sampling

**Figure 6:**
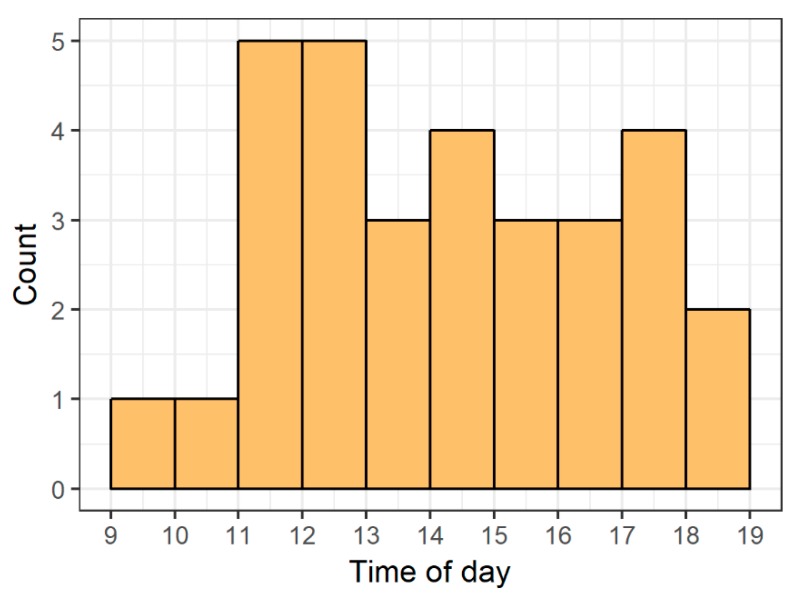
Number of heightened-shoaling episodes by hour of the day. We observed heightened-shoaling at least once in all six tanks and occurring throughout tank-lights on hours, but rarely (only once) in two tanks simultaneously (zebrafish housed in naturalistic tanks; *n* = 6 tanks, 10 zebrafish per tank; 10 days selected to observe from a 6-month period; 31 episodes of heightened-shoaling).

**Figure 7:**
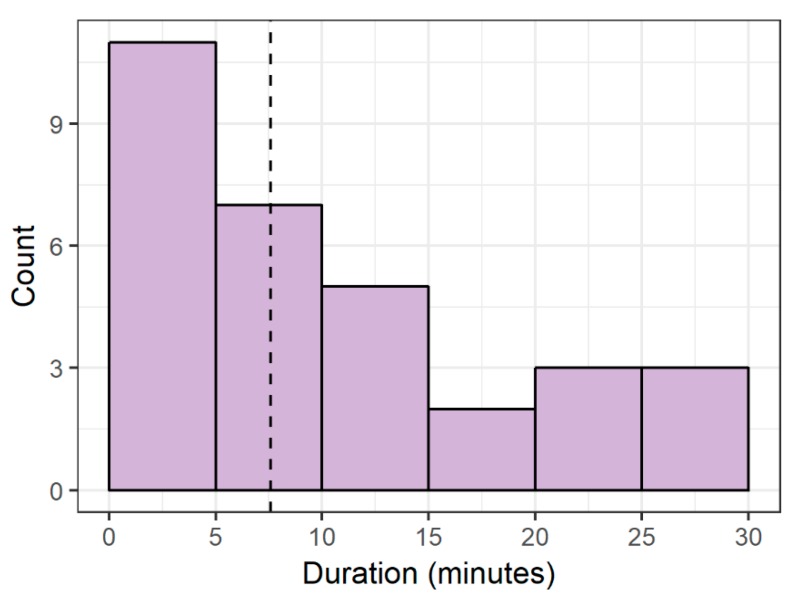
Number of heightened-shoaling episodes by duration (dashed vertical line represents the median heightened-shoaling duration; zebrafish housed in naturalistic tanks; *n* = 6 tanks, 10 zebrafish per tank; 10 days selected to observe from a 6-month period; 31 episodes of heightened-shoaling).

From 10 days of video footage for all 6 tanks (60 tank-days in total), we found 31 instances of heightened-shoaling during tank-light on hours (8:00 h to 18:00 h). We observed this behavior in all 6 tanks, with an average of 5.17 episodes per tank (SD = 4.96). Within the 8:00 h to 18:00 h time-frame, heightened-shoaling was not associated with any particular time (Figure 6). Of the 31 episodes, we only observed 2 beginning within 10 min of each other—the remaining 29 did not coincide with other heightened-shoaling episodes. Episodes lasted for a median time of 7.6 min with a range of 1.3 min to 28.6 min (Figure 7).

## 4. Discussion

Here we find evidence of an affiliative behavior in zebrafish that is seemingly driven and sustained by internal group dynamics. Of the 31 heightened-shoaling episodes evaluated from six tanks over 10 days, only two coincided, suggesting that the episodes were not induced by a room-level event, but instead resulted from internal social affiliation. During heightened-shoaling episodes, which lasted for a median duration of over 7 min, fish were more cohesive, more behaviorally synchronized, and less agonistic than during baseline periods. While we did occasionally observe high group cohesion during baseline and pre-feed times, these instances lasted for brief periods of time (just during a single scan sample) and did not coalesce into heightened-shoaling. As such, heightened-shoaling is categorically different from baseline behavior and best identified by protracted group cohesion (the shortest episode recorded here lasting 1.3 min) along with high participation rates, low agonism, and greater adherence to the horizontal plane. 

While there are some similarities between heightened-shoaling and schooling behavior, there are important distinctions. The characteristic marker of schooling is polarization, that is, the majority of the fish face in the same direction at the same time or show the same movement trajectory [27]. Sometimes schooling groups of fish can be more cohesive than non-schooling groups of fish, but cohesion is not a necessary condition for schooling. Heightened-shoaling, on the other hand, is marked by increased cohesion and increased behavioral synchrony, but not increased polarity *per se*. Specifically, we observed that during heightened-shoaling, the fish are not only substantially more cohesive than normal, they are also more likely to adhere to the horizontal plane, though not necessarily facing in the same direction. Indeed, the polarity during heightened-shoaling could be quite low with approximately half the fish facing directly to the right and half of the fish facing directly to the left. It would be interesting for future research to compare the degree of polarization during heightened-shoaling vs. contexts in which zebrafish are known to engage in schooling behavior [27].

In contrast, zebrafish shoals are defined as a “relatively non-polarized group…held together by social pressures” and are thought to facilitate a range of functions including foraging, spawning, and predator defense [28]. Such social affiliation has been shown to be beneficial to zebrafish. For example, laboratory research has suggested that social isolation can lead to dysfunction later in life [29] and that mere visual contact with conspecifics can serve as a positive reinforcer in a spatial learning task [30].

Studies of wild zebrafish have found absolute differences in shoal cohesion (e.g., nearest neighbor distances) and aggression by geographical location [31], but social dynamics—i.e., how shoal cohesion and other social behaviors may vary across time—have not yet been systematically investigated in detail under unperturbed and naturalistic conditions. The results of the present study relied on the analysis of 600 h of high-definition video collected on fish recorded continuously during daylight hours for 10 days. Such behavioral analysis would be somewhat difficult in the field, in part because zebrafish would be hard to track continuously for such extended periods of time in their natural environments. Thus, the absence of a description of this behavior from the studies done on wild populations of zebrafish is potentially unsurprising and not necessarily indicative that zebrafish do not engage in heightened-shoaling in the wild.

In contrast, zebrafish social behavior has been studied more extensively in standard laboratory environments (reviewed in Reference [32]). The tradition of housing laboratory zebrafish in highly artificial barren tanks may account for the lack of information about heightened-shoaling behavior in the laboratory science literature—laboratory zebrafish are typically housed in small (3 L), empty, and transparent tanks, despite research demonstrating their preference for larger and more complex environments [2,3]. The fish in the present study were housed in tanks that were almost 36 times larger than standard tank size and included three opaque black walls, sloping gravel substrate, artificial plants, and rocks. Previous work has suggested that tank architecture and stocking density can have a large impact on zebrafish social dynamics [33]. To better understand the causes of the onset and occurrence of heightened-shoaling, future studies could investigate the behavioral precursors to heightened-shoaling episodes and which structural features may increase heightened-shoaling frequency including, for example, larger spaces, gravel substrate, sloping substrate, and general complexity in tank design. 

With the present evidence, the valence (positive, negative, neutral) of heightened-shoaling remains somewhat uncertain. However, during heightened-shoaling episodes, we found no evidence of diving behavior, which is commonly associated with distress in zebrafish [24]. Furthermore, heightened-shoaling was characterized by increased behavioral synchrony, which by definition means the individuals were behaving less erratically (it would be impossible to be erratic and coordinated at the same time). Erratic behavior is another behavioral indicator of negative affect in zebrafish [34], providing additional evidence that the behavioral features of heightened-shoaling are inconsistent with negative emotional experience in zebrafish. While more work is needed to distinguish between a neutral and positive emotional valence, the protracted length (some episodes lasting nearly half an hour) and high participation rates within the tank (low average inter-fish distances and universal participation) are indicative of a behavior that is generally attractive and self-reinforcing in nature. Moreover, our results parallel the patterns of certain affiliative behaviors, such as social grooming and social play, that have been linked to positive emotional experience in other species [19,23,35]. Specifically, heightened-shoaling (i) is characterized by increased behavioral synchrony in the absence of signs of distress, (ii) has high rates of participation and sustained durations, and (iii) appears to be driven by internal group dynamics rather than external stimuli. If it is related to positive emotional experience, we would expect future research to find, for example, that agonism would continue to be reduced after the episode ends and that stress reactivity would also be decreased (e.g., lower levels of water-borne cortisol).

With the increased use of fish across all domains of human activity—farming, wild harvest, ornamental aquaria, and science—the welfare of fish, including zebrafish [1], is attracting increased attention [36]. Concurrently, the field of animal welfare has recognized the importance of positive welfare, with the understanding that welfare cannot be achieved by simply removing negative experiences; animals also require choice, control, learning opportunities, and appropriate social affiliation to secure acceptable levels of welfare [12,13,37,38]. As yet, however, very little is known about the intersection of these two trends: Positive welfare in fish. There are many exciting avenues to explore on this topic including, for example, play in fish [39,40]. Our results suggest that heightened-shoaling episodes may be another promising area of inquiry for future investigations into positive emotions in zebrafish.

## 5. Conclusions

Here we find evidence of a previously unreported affiliative behavior in zebrafish: Heightened-shoaling. Compared to baseline behavior, while engaged in heightened-shoaling, fish showed reduced agonism, greater adherence to the horizontal plane (average deviation from horizontal 8.39 degrees, with a 90% coverage interval ranging from 0 to 30 degrees), and tighter shoal cohesion (average inter-fish distances of 8.12 cm, with a 90% coverage interval ranging from 5.05 cm to 12.80 cm). Inter-fish distances during baseline behavior almost never reached such low levels, resulting in a bimodal distribution of inter-fish distances and providing evidence that heightened-shoaling is a distinct behavior. Moreover, during heightened-shoaling, we found no evidence of behaviors that indicate distress in zebrafish (diving behavior or erratic swimming). Scanning video for all instances of heightened-shoaling across ten days for all 6 tanks, we found 31 episodes; they occurred throughout daylight hours and had a median duration of 7.6 min (ranging from 1.3 min to 28.6 min). Of the 31 episodes, only 2 occurred within 10 min of each other, suggesting that the onset of heightened-shoaling is not driven by room-level events. 

In sum, heightened-shoaling is defined by the spontaneous onset of protracted durations of low agonism, high adherence to the horizontal plane, and tight group cohesion, all without signs of distress (i.e., diving or erratic swimming). While the function of heightened-shoaling remains unknown, one possibility is that it facilitates zebrafish communication and/or social bonding, potentially through chemosensory or behavioral signaling, or through changes in hydrodynamic flow and pressure. More work is needed to determine the function and mechanism, but the present research indicates that heightened-shoaling is a candidate for future research on positive emotional behavior in zebrafish and could thereby contribute to our understanding of fish welfare.

## Figures and Tables

**Table 1:** Summary statistics. Zebrafish were observed in naturalistic tanks over a 6-month period (*n* = 6 tanks, 10 zebrafish per tank), with 4 days selected for scan-sampling (producing 180, 180, and 90 scan-sample observation periods for the baseline, pre-feed, and heightened-shoaling periods respectively) and 10 days selected for all-occurrence-sampling (producing 31 heightened shoaling episodes for analyses). X-location and Y-location measured with the origin (0, 0) at the bottom left-hand corner of the tank. **Bold font** indicates significantly different than baseline (multilevel models controlling for repeated sampling of tanks and days, see statistical methods for more details). * Duration was the only behavior collected using all-occurrence sampling methodology and not applicable (NA) to the baseline and pre-feed periods; all other behaviors were collected using scan-sampling methodology.

Behavioral Parameter	Baseline	Pre-Feed	Heightened-Shoaling
X-location			
Mean (Var)	40.84 (447.81) cm	40.38 (518.70) cm	38.38 (64.89) cm
90% Coverage Interval	6.40–74.40 cm	4.80–75.20 cm	24.44–49.60 cm
Y-location			
Mean (Var)	18.02 (47.93) cm	**20.74 (42.48)** cm	16.63 (9.58) cm
90% Coverage Interval	6.00–28.20 cm	7.20–28.20 cm	11.30–21.60 cm
Horizontal-deviation			
Mean (Var)	16.07 (308.30) deg	17.09 (346.13) deg	**8.39 (116.28) deg**
90% Coverage Interval	0.00–45.00 deg	0.00–60.00 deg	0.00–30.00 deg
Inter-fish distances			
Mean (Var)	25.16 (32.48) cm	23.61 (56.90) cm	**8.12 (7.47)** cm
90% Coverage Interval	15.28–33.30 cm	11.05–36.03 cm	5.05–12.80 cm
Agonism			
Probability	0.61	**0.30**	**0.22**
Duration *			
Mean (Var)	*NA*	*NA*	10.88 (70.33) min.
90% Coverage Interval	*NA*	*NA*	2.49–27.07 min.

## Supplementary Materials

The following are available online at http://www.mdpi.com/2076-2615/8/9/152/s1.

## References

[1] Lidster K., Readman G.D., Prescott M.J., Owen S.F. (2017). International survey on the use and welfare of zebrafish *Danio rerio* in research. J. Fish Biol..

[2] Kistler C., Hegglin D., Würbel H., Konig B. (2011). Preference for structured environment in zebrafish (*Danio rerio*) and checker barbs (*Puntius oligolepis*). Appl. Anim. Behav. Sci..

[3] Schroeder P.G., Jones S., Young I.S., Sneddon L.U. (2014). What do zebrafish want? Impact of social grouping, dominance and gender on preference for enrichment. Lab. Anim..

[4] Dawkins M.S. (1990). From an animal’s point of view: Motivation, fitness, and animal welfare. Behav. Brain Sci..

[5] Schroeder P.G., Sneddon L.U. (2017). Exploring the efficacy of immersion analgesics in zebrafish using an integrative approach. Appl. Anim. Behav. Sci..

[6] Speedie N., Gerlai R. (2008). Alarm substance induced behavioral responses in zebrafish (*Danio rerio*). Behav. Brain Res..

[7] Blaser R.E., Chadwick L., McGinnis G.C. (2010). Behavioral measures of anxiety in zebrafish (*Danio rerio*). Behav. Brain Res..

[8] Rey S., Huntingford A.F., Boltaña S., Vargas R., Knowles G.T., Mackenzie S. (2015). Fish can show emotional fever: Stress-induced hyperthermia in zebrafish. Proc. R Soc. B.

[9] Pittman J.T., Lott C.S. (2014). Startle response memory and hippocampal changes in adult zebrafish pharmacologically-induced to exhibit anxiety/depression-like behaviors. Physiol. Behav..

[10] Fulcher N., Tran S., Shams S., Chatterjee D., Gerlai R. (2017). Neurochemical and behavioral responses to unpredictable chronic mild stress following developmental isolation: the Zebrafish as a model for major depression. Zebrafish.

[11] Kok B.E., Coffey K.A., Cohn M.A., Catalino L.I., Vacharkulksemsuk T., Algoe S.B., Brantley M., Fredrickson B.L. (2013). How positive emotions build physical health: Perceived positive social connections account for the upward spiral between positive emotions and vagal tone. Psychol. Sci..

[12] Yeates J.W., Main D.C.J. (2008). Assessment of positive welfare: A review. Vet. J..

[13] Mellor D.J. (2016). Moving beyond the “Five Freedoms” by Updating the “Five Provisions” and Introducing Aligned “Animal Welfare Aims”. Animals.

[14] Kittilsen S. (2013). Functional aspects of emotions in fish. Behav. Processes.

[15] Hughes R.N. (1997). Intrinsic exploration in animals: Motives and measurement. Behav. Processes.

[16] Graham C., von Keyserlingk M.A.G., Franks B. (2018). Free-choice exploration increases affiliative behaviour in zebrafish. Appl. Anim. Behav. Sci..

[17] Faustino A.I., Tacão-Monteiro A., Oliveira R.F. (2017). Mechanisms of social buffering of fear in zebrafish. Sci. Rep..

[18] Rault J.L. (2012). Friends with benefits: Social support and its relevance for farm animal welfare. Appl. Anim. Behav. Sci..

[19] Špinka M. (2012). Social dimension of emotions and its implication for animal welfare. Appl. Anim. Behav. Sci..

[20] Soares M.C., Oliveira R.F., Ros A.F.H., Grutter A.S., Bshary R. (2011). Tactile stimulation lowers stress in fish. Nat. Commun..

[21] Bshary R., Wurth M. (2001). Cleaner fish Labroides dimidiatus manipulate client reef fish by providing tactile stimulation. Proc. Biol. Sci..

[22] Spinka M., Newberry R.C., Bekoff M. (2001). Mammalian play: Training for the unexpected. Q. Rev. Biol..

[23] Boissy A., Boissy A., Manteuffel G., BakJensen M., Moe R.O., Spruijt B., Keeling L.J., Winckler C., Forkman B., Dimitrov I. (2007). Assessment of positive emotions in animals to improve their welfare. Physiol. Behav..

[24] Blaser R.E., Rosemberg D.B. (2012). Measures of anxiety in zebrafish (*Danio rerio*): Dissociation of black/white preference and novel tank test. PLoS ONE.

[25] Gelman A., Hill J. (2006). Data Analysis Using Regression and Multilevel/Hierarchical Models.

[26] Snijders T.A.B., Bosker R.J. (2012). Multilevel Analysis: An Introduction to Basic and Advanced Multilevel Modeling.

[27] Miller N., Gerlai R. (2012). From Schooling to Shoaling: Patterns of Collective Motion in Zebrafish (*Danio rerio*). PLoS ONE.

[28] Pitcher T.J. (1983). Heuristic definitions of fish shoaling behaviour. Anim. Behav..

[29] Collymore C., Tolwani R.J., Rasmussen S. (2015). The Behavioral Effects of Single Housing and Environmental Enrichment on Adult Zebrafish (*Danio rerio*). J. Am. Assoc. Lab. Anim. Sci..

[30] Al-Imari L., Gerlai R. (2008). Sight of conspecifics as reward in associative learning in zebrafish (*Danio rerio*). Behav. Brain Res..

[31] Suriyampola P.S., Shelton D.S., Shukla R., Roy T., Bhat A., Martins E.P. (2016). Zebrafish Social Behavior in the Wild. Zebrafish.

[32] Graham C., von Keyserlingk M.A.G., Franks B. (2018). Zebrafish welfare: Natural history, social motivation and behaviour. Appl. Anim. Behav. Sci..

[33] Shelton D.S., Price B.C., Ocasio K.M., Martins E.P. (2015). Density and group size influence shoal cohesion, but not coordination in zebrafish (*Danio rerio*). J. Comp. Psychol..

[34] Quadros V.A., Silveira A., Giuliani G.S., Didonet F., Silveira A.S., Nunes M.E., Silva T.O., Loro V.L., Rosemberg D.B. (2016). Strain- and context-dependent behavioural responses of acute alarm substance exposure in zebrafish. Behav. Processes.

[35] Held S.D.E., Špinka M. (2011). Animal play and animal welfare. Anim. Behav..

[36] Brown C. (2015). Fish intelligence, sentience and ethics. Anim. Cogn..

[37] Spinka M., Wemelsfelder F., Appleby M.C., Olsson I.A.S., Galindo F. (2018). Environmental challenge and animal agency. Animal Welfare.

[38] Franks B., Mench J.A. (2017). Cognition as a cause, consequence, and component of welfare. Advances in Agricultural Animal Welfare: Science and Practice.

[39] Burghardt G.M. (2005). The origins of vertebrate play: Fish that leap, juggle, and tease. The Genesis of Animal Play: Testing the Limits.

[40] Fagen R. (2017). Salmonid Jumping and Playing: Potential Cultural and Welfare Implications. Animals.

